# One Drop App With an Activity Tracker for Adults With Type 1 Diabetes: Randomized Controlled Trial

**DOI:** 10.2196/16745

**Published:** 2020-09-17

**Authors:** Chandra Y Osborn, Ashley Hirsch, Lindsay E Sears, Mark Heyman, Jennifer Raymond, Brian Huddleston, Jeff Dachis

**Affiliations:** 1 Informed Data Systems Inc New York, NY United States; 2 Lirio Nashville, TN United States; 3 Sarah Cannon Research Institute Nashville, TN United States; 4 Department of Psychiatry University of California San Diego San Diego, CA United States; 5 Division of Endocrinology Department of Pediatrics University of Southern California Los Angeles, CA United States

**Keywords:** diabetes, type 1 diabetes, digital therapy, mobile app, coaching, glucometer, activity tracker

## Abstract

**Background:**

In 2017, mobile app support for managing diabetes was available to 64% of the global population of adults with diabetes. One Drop’s digital therapeutics solution includes an evidence-based mobile app with global reach, a Bluetooth-connected glucometer, and in-app coaching from Certified Diabetes Educators. Among people with type 1 diabetes and an estimated hemoglobin A_1c_ level≥7.5%, using One Drop for 3 months has been associated with an improved estimated hemoglobin A_1c_ level of 22.2 mg/dL (–0.80%). However, the added value of integrated activity trackers is unknown.

**Objective:**

We conducted a pragmatic, remotely administered randomized controlled trial to evaluate One Drop with a new-to-market activity tracker against One Drop only on the 3-month hemoglobin A_1c_ level of adults with type 1 diabetes.

**Methods:**

Social media advertisements and online newsletters were used to recruit adults (≥18 years old) diagnosed (≥1 year) with T1D, naïve to One Drop’s full solution and the activity tracker, with a laboratory hemoglobin A_1c_ level≥7%. Participants (N=99) were randomized to receive One Drop and the activity tracker or One Drop only at the start of the study. The One Drop only group received the activity tracker at the end of the study. Multiple imputation, performed separately by group, was used to correct for missing data. Analysis of covariance models, controlling for baseline hemoglobin A_1c_, were used to evaluate 3-month hemoglobin A_1c_ differences in intent-to-treat (ITT) and per protocol (PP) analyses.

**Results:**

The enrolled sample (N=95) had a mean age of 41 (SD 11) years, was 73% female, 88% White, diagnosed for a mean of 20 (SD 11) years, and had a mean hemoglobin A_1c_ level of 8.4% (SD 1.2%); 11% of the participants did not complete follow up. Analysis of covariance assumptions were met for the ITT and PP models. In ITT analysis, participants in the One Drop and activity tracker condition had a significantly lower 3-month hemoglobin A_1c_ level (mean 7.9%, SD 0.60%, 95% CI 7.8-8.2) than that of the participants in the One Drop only condition (mean 8.4%, SD 0.62%, 95% CI 8.2-8.5). In PP analysis, participants in the One Drop and activity tracker condition also had a significantly lower 3-month hemoglobin A_1c_ level (mean 7.9%, SD 0.59%, 95% CI 7.7-8.1) than that of participants in the One Drop only condition (mean 8.2%, SD 0.58%, 95% CI 8.0-8.4).

**Conclusions:**

Participants exposed to One Drop and the activity tracker for the 3-month study period had a significantly lower 3-month hemoglobin A_1c_ level compared to that of participants exposed to One Drop only during the same timeframe. One Drop and a tracker may work better together than alone in helping people with type 1 diabetes.

**Trial Registration:**

ClinicalTrials.gov NCT03459573; https://clinicaltrials.gov/ct2/show/NCT03459573.

## Introduction

Diabetes is common, costly, and can have serious consequences. An estimated 30 million people in the United States are living with diabetes, 1.3 million of whom have type 1 diabetes (T1D) [1] indicated by the pancreas producing little or no insulin. Approximately US $327 billion is spent annually to treat diabetes, complications from diabetes, and employees’ losses in productivity [2]. Moreover, at least 11% of annual US deaths can be attributed to a diabetes complication [3]. A hemoglobin A_1c_ level of <7% reduces the risk of developing diabetes complications [4,5], but can be hard to achieve for a variety of reasons [6]. People with T1D achieve “at goal” blood glucose with a combination of insulin therapy, carbohydrate monitoring, blood glucose monitoring, and physical activity [7,8]. Frequent, painful insulin injections and finger pricks have historically made it difficult to administer insulin and check blood glucose as recommended [9]. However, digital advances in the last 20 years have made administering insulin (ie, via insulin pumps) and monitoring blood glucose (ie, via continuous glucose monitors) much easier [10,11]. Other consumer technologies such as health apps, digital therapies, and wearable activity trackers can aid in the management of T1D, making it easier and more convenient to perform and monitor self-care activities [12].

Consumer apps and activity trackers can help people with T1D meet their glycemic targets [13-16]. Among adolescents with T1D, using an activity tracker has been associated with being more physically active and having an improved average time in-range blood glucose level after a 3-month period [13]. Among children with T1D wearing activity trackers with health care providers remotely monitoring their tracker data (and other data) [14], quality of life and hemoglobin A_1c_ improved after 3 months [14]. However, 3-month hemoglobin A_1c_ benefits among adults with T1D using trackers independent of remote monitoring is unknown.

Adults with T1D can use the One Drop mobile smartphone app with or without activity trackers (eg, Apple Watch [17,18]) [16]. One Drop’s app reads and displays activity data from trackers and other devices and is rated among the top three diabetes apps in the world [19]. The One Drop Chrome Bluetooth-connected meter syncs and displays blood glucose readings in the app. One Drop’s Certified Diabetes Educators (CDE “coaches”) remotely monitor user data and offer in-app education, strategies, and support. In observational studies, people with T1D or type 2 diabetes using One Drop’s app on Apple Watch averaged a –1.2% to –1.3% absolute estimated hemoglobin A_1c_ improvement [17,18].

Studies consistently associate using One Drop’s solution with improved estimated hemoglobin A_1c_, but none of these studies used a randomized controlled trial design or included people with T1D using an activity tracker or smartwatch. Therefore, we conducted a prospective randomized controlled trial with adults with T1D to evaluate the 3-month effect of using One Drop and a new-to-market activity tracker on hemoglobin A_1c_.

## Methods

### Study Design

Solutions IRB, a private Institutional Review Board (IRB) registered with HHS #IRB00008523 and accredited by the Association of Human Research Protection Programs, approved all study procedures prior to recruiting participants. The study design was a pragmatic, parallel group, randomized controlled trial. Study personnel used a block randomization scheme of 100 groups of two randomization blocks to randomize participants to one of two conditions: (1) One Drop’s digital therapeutics solution (ie, the mobile app, in-app coaching, Bluetooth-connected meter with a 3-month supply of test strips) and an activity tracker at the start of the intervention period or (2) One Drop at the start of the intervention period and an activity tracker after completing 3-month follow-up measures. Participants and study personnel were unblinded to the condition assignment. Study personnel did not tell participants which condition was the intervention of interest and which one was the comparator, but participants may have inferred this on their own. Study instructions, consent, Health Insurance Portability and Accountability Act (HIPAA) authorization forms, and self-reported surveys were self-administered online using HIPAA-compliant surveys and forms. Participants used a mail-in hemoglobin A_1c_ test to self-collect and supply two blood specimens. Study personnel provided virtually disseminated instruction and support (via phone and email) to remotely eligible participants in their respective study conditions. Only participants accessing all intervention components were considered to be enrolled in the trial.

### Recruitment

Facebook advertisements and One Drop’s email list of noncustomers (ie, people never having used a One Drop meter, testing supplies, or coach) remotely recruited potential participants from March through May of 2018. Online advertisements and email messages briefly described study eligibility (eg, diagnosis of T1D), study scope (eg, 3-month duration), and asked people interested in the study to click a link to obtain in-depth information about the study and complete an online, HIPAA-compliant survey to self-screen for initial eligibility.

### Eligibility

Initially eligible individuals met screening survey criteria. They self-reported an age of 18-75 years, had a valid US mailing address, a diagnosis of T1D for ≥1 year, were not currently participating in a diabetes education or coaching program, were not pregnant or planning to become pregnant, were using an Android or iOS smartphone, and had never used the activity tracker or One Drop (no app activity, 7-day trial, testing supply subscription, or coaching). An application programming interface (ie, a software intermediary for transferring data from one application [One Drop’s database] to another [an Excel spreadsheet]) automatically and objectively checked whether respondents had previously used any aspect of One Drop. Any participant not meeting initial eligibility criteria was notified of this on the screening survey’s final landing page and thanked for their interest in the study.

People who self-screened as eligible followed a different path. They landed on an electronic IRB-approved consent form and HIPAA authorization form, requiring review and signature. All respondents were invited to contact study personnel to receive a verbal explanation of the forms or have any study-related questions answered. Once respondents electronically signed both forms, they landed on an online, HIPAA-compliant baseline survey. Upon completing the baseline survey, DTI Laboratories, Inc. shipped an AccuBase hemoglobin A_1c_ test kit to each respondent’s mailing address. Study personnel provided written, illustrated, and video hemoglobin A_1c_ test kit instructions and offered over-the-phone help in collecting a blood sample. The participants returned blood samples to the lab in a preaddressed and prestamped box. The lab processed each sample and uploaded results into a HIPAA-compliant online portal. Study personal reviewed each result to determine hemoglobin A_1c_ eligibility.

Participants with a hemoglobin A_1c_ level≥7% were considered eligible for the study, randomized to one of the two conditions, and notified of their hemoglobin A_1c_ test result and condition assignment. People deemed ineligible (ie, hemoglobin A_1c_<7%) were also notified of their hemoglobin A_1c_ test result, told they did not meet the hemoglobin A_1c_ criterion for participation, and were thanked for their interest in the study.

### Data Collection and Procedures

#### Baseline Data Collection

The baseline survey collected demographic and diabetes information and responses to other self-report measures. The hemoglobin A_1c_ test determining study eligibility also served as the participants’ measure of baseline hemoglobin A_1c_.

#### Randomization

We used an online randomizer to block-randomize 100 groups of two randomization blocks to randomize participants to receive One Drop’s digital therapeutics solution and an activity tracker at the start of the intervention period or One Drop at the start of the intervention period and an activity tracker after completing follow-up measures.

#### One Drop

The digital therapeutics solution includes the accurate Food and Drug Administration (FDA)-approved One Drop Chrome Bluetooth-connected glucometer [20] and testing supplies, One Drop mobile smartphone and smartwatch app, and One Drop coaching programs. One Drop coaches are real-life CDEs providing the first digitally delivered diabetes education program accredited by the American Diabetes Association. Users of One Drop’s digital therapeutics solution have 24/7 in-app access to their personal CDE coach who answers questions, offers tips and advice, and provides practical and emotional support and accountability for daily self-care. One Drop’s evidenced-based app [21] is available on iOS, Android, watchOS, and Amazon’s Alexa and has been downloaded in every country in the world. Features include reminders to perform and track self-care, a “Community” section to bolster normative support, and education and skills training via the dynamic “Newsfeed” section along with the coaching chat section and programming content. Data reports can be viewed in the app, printed, and emailed.

As is the case with all apps, occasional minor bug fixes are typical; however, none of these resulted in major system failures or downtimes during the study period.

#### Activity Tracker

The wrist-worn device tracks activity and swimming, monitors heart rate, includes a built-in GPS, real-time statistics (eg, pace and distance), phone-free music to exercise with, and personalized workouts. With each workout, the software learns about a user’s fitness level, makes personalized recommendations, and gives dynamic feedback. Third-party app developers such as One Drop can make device-compatible apps. One Drop’s app on the device is an at-a-glance display of the last minutes of activity, grams of carbohydrates last consumed, last blood glucose reading, and last medications taken.

Once randomized to a condition, eligible participants received an email message containing their condition assignment, a series of instructions, and a unique verification code. The email message instructed participants to first download the One Drop mobile app on iOS or Android with embedded links to both formats for direct access. Next, participants were instructed to open the One Drop app, create an account, and enter their unique coaching verification code (from the email). Finally, participants were given a link to One Drop’s online store and instructed to trigger a no-cost shipment of the activity tracker, One Drop meter, and testing supplies for the 3-month study period. When needed, study personnel assisted participants with completing these steps via a phone call or email exchange.

Attempts were made to keep study personnel blinded to condition. Randomization and condition assignment occurred separately with different researchers. A single researcher responded to participants’ technical and research-related questions. All study procedures were digitally accessible. Most procedures were automated and self-administered, maintaining limited researcher touch and balance between groups. Furthermore, researchers and participants were separated the vast majority of the time.

#### One Drop and Activity Tracker at Study Start

Participants assigned to the One Drop and activity tracker condition were exposed to One Drop’s comprehensive digital therapeutics solution and an integrated activity tracker at the start of the study. First, participants were mailed the activity tracker, One Drop meter, and testing supplies. Participants were also emailed “how-to” videos, written instructions, and offered study personnel support for setting up their devices and adjusting their smartphone settings to view activity data in the One Drop app. How-to videos included how to set up their activity tracker on a smartphone or computer, automatically record exercises, sync data with other health apps, personalize notifications, and perform other customizations. Written instructions were supplied on how to directly integrate the tracker with One Drop to view tracker activity data (minutes and steps) in the One Drop app and view One Drop data on the tracker’s clockface. Study personnel were also available 24/7 to answer participants’ technical questions via email, text message, and phone calls.

Upon receipt of the activity tracker and One Drop’s meter/strips, participants were instructed to connect with their coach (a real-life CDE) in the One Drop app, download the tracker-compatible One Drop app, and link their One Drop and activity tracker accounts. Finally, participants were instructed to use One Drop’s app on their smartphone and activity tracker, One Drop’s meter/strips, and in-app coaching “as needed” for the 3-month study period. Participants could initiate two-way communication with their coach about a wide range of diabetes self-care topics. For physical activity, topics may include, but are not limited to, those related to a participant’s tracked activity (visible on the coach’s dashboard), reasonable goal setting around minutes of activity or steps walked per day/week, or how to manage blood glucose levels before and after a bout of exercise.

#### One Drop and Activity Tracker at Study End

Participants assigned to the One Drop only condition were mailed the One Drop meter and testing supplies. The activity tracker was shipped after completing the follow-up survey and hemoglobin A_1c_ test. Participants connected with their One Drop coach via the One Drop app, and were instructed to use the app, meter, and in-app coach “as needed” for the 3-month study period.

#### Follow-Up Data Collection

After 3 months, participants in both conditions received an initial email and then a series of reminder emails instructing them to complete an online, HIPAA-compliant follow-up survey hyperlinked in the email. After participants completed this survey, DTI Laboratories, Inc. mailed a hemoglobin A_1c_ test kit to assess participants’ 3-month hemoglobin A_1c_. Again, study personnel sent instructions in various formats along with study contact information to aid with collecting a blood sample. Participants returned blood samples in a preaddressed and prestamped box. The lab processed each sample and uploaded results into the HIPAA-compliant online portal.

Study personal reviewed follow-up hemoglobin A_1c_ results, shared results with each participant, thanked them for their participation, and sent a discount code for a monthly and annual One Drop subscription. After participants in the One Drop only condition completed the follow-up survey and hemoglobin A_1c_ test, study personnel shipped their activity tracker to their mailing address. There were no methodological changes during the study period.

#### Compensation

Participant compensation included 3 free months of One Drop testing supplies and in-app coaching, and a free One Drop Chrome Bluetooth-connected meter and activity tracker to keep beyond the study period.

### Measures

#### Demographic Characteristics

The baseline survey collected self-reported age, gender, race/ethnicity, education, annual income, and health insurance status.

#### Health Status

Health status information included self-reported number of years since a diabetes diagnosis and BMI.

#### Digital Health History

At baseline, participants self-reported whether or not they had ever used a blood glucose monitoring device (finger stick, continuous glucose monitor, flash monitor), a diabetes app, the new-to-market wearable tracker being used in the study, or any other wearable tracker to manage their health. Response options were “yes” or “no.”

#### Digital Usability

At follow up, participants self-reported on a 7-point Likert scale ranging from “extremely hard” to “extremely easy” how hard to easy it was to use One Drop’s meter, diabetes app, and in-app coaching. Participants in the One drop and activity tracker condition also self-reported how “extremely hard” to “extremely easy” it was to use the new-to-market activity tracker included in the study.

#### Digital Engagement and Attrition

At follow up, participants self-reported on a 7-point Likert scale ranging from “never” to “always” how often they used One Drop’s meter, diabetes app, and in-app coaching when needing to check blood glucose, manage diabetes, or get help with diabetes, respectively. We then verified self-reported One Drop engagement with objectively collected data through the One Drop app. Participants in the One Drop and activity tracker condition also self-reported how often they used the new-to-market activity tracker during the 3-month study period.

#### Hypoglycemic Events

Hypoglycemia (ie, an extremely low blood glucose level requiring assistance) is a rate-limiting factor in the management of T1D and optimization of blood glucose. We accounted for the occurrence of hypoglycemia during the study period by asking all participants a single question in the 3-month follow-up survey: “In the past 3 months, how many times have you had a low blood sugar requiring help?” Responses were based on counts.

#### Glycated Hemoglobin A_1c_

Hemoglobin A_1c_ was measured twice. Self-administered AccuBase A_1c_ Mail-In Test Kits (DTI Laboratories, Inc., Thomasville, GA, USA) were used to assess baseline and 3-month hemoglobin A_1c_ levels. This test is FDA-approved, certified by the National Glycohemoglobin Standardization Program, Clinical Laboratory Improvement Amendments-waived, and a highly accurate assessment of hemoglobin A_1c_ used in randomized and nonrandomized trials [22,23]. It is a nonfasting, finger stick, whole blood mail-in test. Upon supplying a blood sample, specimens are processed at a central lab.

### Statistical Analyses

Statistical analyses followed the Consolidated Standards of Reporting Trials (CONSORT) guidelines for randomized trials. Data were analyzed using SPSS version 24. Sample data at baseline overall and separately by group are described as means (SD) or counts (n, %) as appropriate. We followed CONSORT guidelines for testing and treatment of baseline group differences [24].

Counts characterized any engagement with One Drop during the study period, and for completing the study overall and by condition assignment. Two Chi-square tests were used to assess One Drop engagement and to assess study completion differences between groups.

Descriptive statistics were also used to characterize baseline digital health history, follow-up digital usability and engagement, study attrition, and the number of hypoglycemic events experienced during the study period overall and by study condition. Chi-square tests and Mann Whitney *U* tests were used to assess these particular baseline and follow-up group differences.

Multiple imputation was used to correct for missing data [25] on income (n=4) and follow-up hemoglobin A_1c_ (n=10). In both groups, variables used to impute included nonmissing age, gender race/ethnicity, education, insurance status, diabetes duration, baseline BMI and hemoglobin A_1c_, and available data on income. Data were imputed separately by study condition. Imputed data were constrained by condition-specific minimum and maximum values. There were 20 imputations per condition. Data were merged prior to conducting intent-to-treat (ITT) and per protocol (PP) analyses.

Analysis of covariance (ANCOVA) models [26] were used to test the group effect on follow-up hemoglobin A_1c_ controlling for baseline hemoglobin A_1c_. For noncrossover, parallel group randomized controlled trials, CONSORT guidelines recommend reporting results from ITT and PP analyses [27,28]. ITT analysis preserves baseline condition assignment and avoids overestimating group effects [29]. In contrast, PP analysis may exaggerate group effects by including only participants receiving the allocated intervention and completing the study as intended [30,31]. In pragmatic trials, the appropriate reporting of both results can aid with scientific and clinical interpretation [32]. We examined ANCOVA assumptions before conducting ANCOVA models testing 3-month hemoglobin A_1c_ group differences.

## Results

### Participants

**Figure 1:**
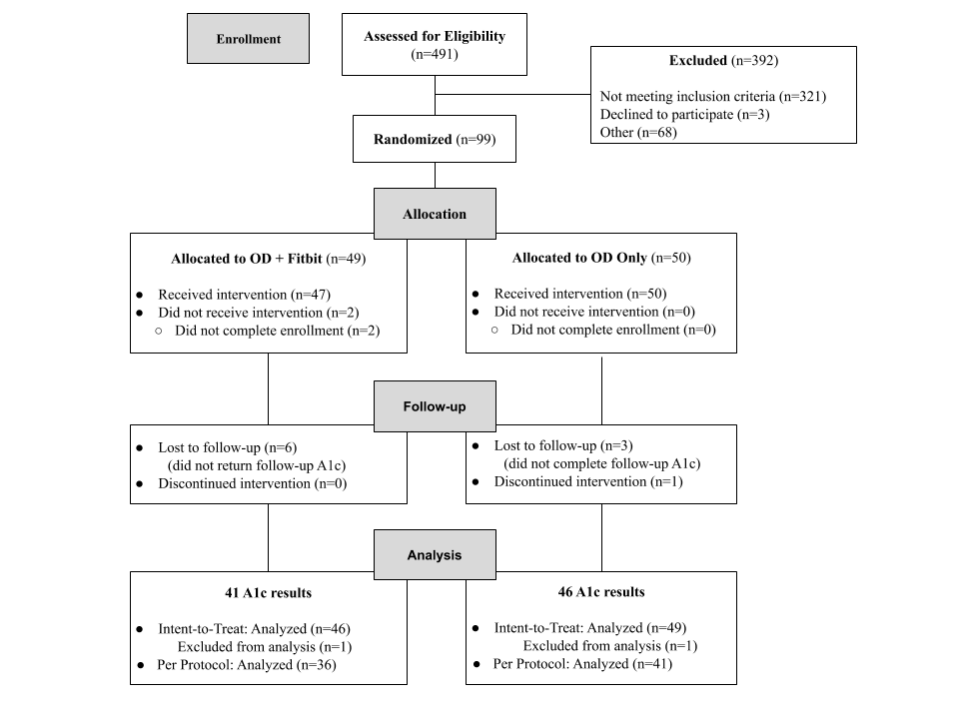
Study flow diagram. OD: One Drop; A_1c_: hemoglobin A_1c_.

Recruitment and enrollment occurred from March through May of 2018, and the last follow up was in August 2018. As shown in the CONSORT diagram (see Figure 1), 491 people self-screened for initial eligibility; 129 screened as initially eligible, completed the informed consent, HIPAA authorization, and the baseline survey, and were shipped a hemoglobin A_1c_ test kit. A total of 112 people returned the kit with a blood sample; 99 people satisfied the eligibility criterion of hemoglobin A_1c_≥7% and were randomized to the One Drop and activity tracker condition or One Drop only condition, 97 of whom received the intervention, defined as creating a One Drop account and enrolling in One Drop coaching during the study period. Study participation resulted in no reported harm, unintended effects, or adverse events. There were also no privacy breaches, severe technical problems, or unexpected/unintended incidents during the study.

Quality assurance efforts identified two participants ineligible for the study (ie, one participant in each condition had used One Drop before) who were excluded from all analyses, resulting in 95 participants for ITT analysis and 77 participants for PP analysis. Participants in the PP analysis used One Drop (app, meter, and/or coaching) at least once during the study period and provided a follow-up hemoglobin A_1c_ blood specimen.

### Digital Health History

All eligible participants self-reported current smartphone utilization. At baseline, all enrolled participants self-reported having previously used a blood glucose monitoring device (eg, finger stick, continuous glucose monitor, flash device), but also reported that they had never used a diabetes app to manage their health or the new-to-market tracker used in the study. However, 61% (58/95) had used some type of wearable device to manage their health prior to the study. Prior experience with a wearable health device did not differ between groups (χ^2^_1_=0.77, *P*=.38).

Among participants that reported on the ease of use or usability of One Drop at follow up, 98% (88/90) reported One Drop’s meter was easy to use, 83% (75/90) reported the app was easy to use, and 75% (67/89) reported in-app coaching was easy to use. The usability of One Drop was comparable between groups (*P*=.49 for the meter, *P*=.48 for the app, and *P*=.40 for in-app coaching). For participants in the One Drop and activity tracker condition, 95% (40/42) reported the activity tracker was easy to use.

### Digital Engagement and Attrition

Among participants that reported on their utilization of One Drop at follow up, 98% (88/90) reported using One Drop’s meter, 80% (72/90) reported using the app, and 38% (34/89) reported using in-app coaching half the time or more during the trial to check blood glucose, manage diabetes, or get help with diabetes, respectively. Self-reported use of One Drop during the trial was comparable between groups (*P*=.99 for the meter, *P*=.66 for the app, and *P*=.11 for in-app coaching).

According to objectively collected One Drop user data, 92% (87/95) of all participants used two or more parts of One Drop’s 3-part solution during the study. No participant used only one part. One Drop utilization was comparable between groups (χ^2^_2_=0.45, *P*=.80).

Among participants in the One Drop and activity tracker condition, 100% (42/42) reported using the tracker half the time or more during the study, 90% (38/42) of whom used it always or almost always during that time.

Additionally, 88% (84/95) of all participants completed the study. Study completion did not differ by condition assignment (χ^2^_1_=0.04, *P*=.83).

### Hypoglycemic Events

The Mann-Whitney *U* test suggested a trending difference in the number of hypoglycemic events experienced during the study period between the One Drop and activity tracker group (n=42) and the One Drop only group (n=47). The One Drop and activity tracker group (1.64, SD 3.05) tended to report fewer hypoglycemic events during the study period compared to the One Drop only group (2.98, SD 4.92) (*P*=.08).

### ITT Analysis

Descriptive statistics were used to summarize the sample characteristics in the ITT analysis (N=95). The sample was about 40 years old on average, and the majority were female, Caucasian/White race, had at least some college education, an annual household income ≥US $50,000, and were overweight or obese. The sample had received a T1D diagnosis about 20 years ago on average with an average baseline hemoglobin A_1c_ of 8.41% (Table 1).

Twenty pooled ANCOVA models were used to test statistical assumptions on 20 sets of imputed data (N=95; One Drop and activity tracker n=46, One Drop only n=49). Both the homogeneity of regression slopes (*F*_pooled1_=1.06, *P*_pooled_<.42) and homogeneity of variance (*F*_pooled1_=2.93, *P*_pooled_<.11) assumptions were met. Twenty pooled ANCOVA models without the interaction term on 20 sets of imputed data revealed a significant main effect for baseline hemoglobin A_1c_ (*F*_pooled1_=227.99, *P*<.001) and a significant main effect for condition assignment (*F*_pooled1_=10.28, *P*<.001, ηp²=.10, observed power=0.87). Follow-up hemoglobin A_1c_ varied by group. After the 3-month study period, participants in the One Drop and activity tracker condition had a significantly lower hemoglobin A_1c_ level (mean 7.9%, SD 0.60%, 95% CI 7.8-8.2) than participants in the One Drop only condition (mean 8.4%, SD 0.62%, 95% CI 8.2-8.5).

**Table 1:** Characteristics of intent-to-treat participants overall and by group.

Characteristic				All Participants (N=95)		One Drop + tracker (n=46)		One Drop only (n=49)
**Demographic characteristics**								
	Age (years), mean (SD)		40.9 (10.7)		41.1 (9.7)		40.7 (11.6)	
	**Gender, n (%)**							
		Female	69 (73)		34 (74)		35 (71)	
		Male	26 (27)		12 (26)		14 (29)	
	**Race/ethnicity, n (%)**							
		Caucasian/White	84 (88)		43 (94)		41 (84)	
		Hispanic/Latino	5 (5)		1 (2)		4 (8)	
		African American/Black	3 (3)		1 (2)		2 (4)	
		Asian	0 (0)		0 (0)		0 (0)	
		American Indian/Alaskan Native	2 (2)		0 (0)		2 (4)	
		Native Hawaiian/Pacific Islander	1 (1)		1 (2)		0 (0)	
	Education years, mean (SD)		14.6 (2.2)		14.4 (2.2)		14.9 (2.1)	
	**Education level, n (%)**							
		Below high school	4 (4)		2 (4)		2 (4)	
		High school graduate/GED^a^	18 (19)		11 (24)		7 (14)	
		Some college	33 (35)		18 (39)		15 (31)	
		College graduate	15 (16)		4 (9)		11 (22)	
		Graduate school	25 (26)		11 (24)		14 (29)	
	**Annual income (USD)^b^, n (%)**							
		<25,000	12 (13)		7 (15)		5 (11)	
		25,000-50,000	26 (27)		13 (28)		13 (29)	
		50,000-100,000	35 (37)		21 (46)		14 (31)	
		>100,000	18 (19)		5 (11)		13 (29)	
**Health status**								
	**Health insurance, n (%)**							
		Yes	89 (94)		45 (98)		44 (90)	
		No	6 (6)		1 (2)		5 (10)	
	Diabetes duration (years), mean (SD)		20.3 (11.5)		21.6 (12.1)		19.1 (10.9)	
	BMI, mean (SD)		30.1 (6.6)		30.6 (7.7)		29.6 (5.4)	
	**BMI category, n (%)**							
		Underweight, BMI<18.5	1 (1)		1 (2)		0 (0)	
		Normal weight, BMI 18.5-24.9	18 (19)		10 (22)		8 (168)	
		Overweight, BMI 25-29.9	32 (34)		12 (26)		20 (41)	
		Obese I, BMI 30-34.9	26 (27)		12 (26)		14 (29)	
		Obese II, BMI 35-39.9	9 (10)		5 (11)		4 (8)	
		Morbidly obese, BMI≥40	9 (10)		6 (13)		3 (6)	
	Hemoglobin A_1c_ (%), mean (SD)		8.4 (1.2)		8.5 (1.9)		8.3 (1.9)	

^a^GED: General Educational Development test.

^b^N=91.

### PP Analysis

A single ANCOVA model was run on the complete data of participants who used One Drop during the study period and provided a 3-month hemoglobin A_1c_ blood specimen (N=77; One Drop and activity tracker n=36, One Drop only n=41). Once again, the ANCOVA assumptions of homogeneity of regression slopes (*F*_1_=153.3, *P*<.001) and homogeneity of variance (*F*_1_=5.36, *P*<.02) were met.

An ANCOVA model without the interaction term revealed a significant main effect for baseline hemoglobin A_1c_ (*F*_1_=153.3, *P*<.001) and a significant main effect for condition assignment (*F*_1_=5.36, *P*<.02, ηp²=.07, observed power=0.63). Follow-up hemoglobin A_1c_ varied by group. Participants who followed protocol during the 3-month study period had a significantly lower hemoglobin A_1c_ level if they were in the One Drop and activity tracker condition (mean 7.9%, SD 0.59%, 95% CI 7.7-8.1) than if they were in the One Drop only condition (mean 8.2%, SD 0.58%, 95% CI 8.0-8.4).

## Discussion

### Principal Findings

This is the first randomized controlled trial evaluating One Drop with an activity tracker vs One Drop alone on the 3-month hemoglobin A_1c_ of adults with T1D. Participants exposed to One Drop and the tracker for the 3-month study period had a significantly lower 3-month hemoglobin A_1c_ compared to that of participants exposed to One Drop only during the same timeframe. Results were consistent in ITT analyses on imputed data and PP analyses on complete data (ie, among participants using all aspects of One Drop who also provided a follow-up hemoglobin A_1c_ value). Moreover, there was a trend of fewer hypoglycemic events experienced during the study period among participants in the One Drop and activity tracker condition relative to those in the One Drop only condition.

Consistent with behavior change theories and their empirical validations [33,34], self-care improvements may have been a mechanism by which the 3-month hemoglobin A_1c_ was better for participants in the One Drop and activity tracker condition than in the One Drop only condition. People tracking their activity experience small but significant improvements in spontaneous lifestyle activity and weight loss [35]. Additional research is needed to determine if using One Drop and an activity tracker improves physical activity, weight, and, in turn, hemoglobin A_1c_.

Using hardware and software to track self-care makes people aware of their activity but may not sufficiently engage and activate them [36]. Being female or overweight/obese has been associated with using an activity tracker [37]. The trial’s predominantly female (73%) and overweight/obese (80%) sample may have been uniquely engaged and activated by using an activity tracker with One Drop. Therefore, these results may not generalize to adults with T1D who are male or have a “normal” BMI.

### Strengths and Limitations

There are study strengths and limitations to note. Prior One Drop studies have been limited by a single group, pre-post design, self-selection, and prior exposure to One Drop as an alternative explanation for the results. This trial’s randomized design addresses some of these limitations while highlighting the benefits of activity trackers for people with T1D using One Drop.

This trial was “pragmatic” [38]. That is, the study procedures were conducted remotely in the context of participants’ everyday lives. Pragmatic trials maximize the applicability and generalizability of the findings but also make study data open to accuracy concerns. For instance, a person other than the participant might have completed the mail-in hemoglobin A_1c_ test. A real-world trial also introduces more confounding variables. For example, participants may have used other wearable devices or health apps during the study period.

People with diabetes, payers, and manufacturers want to know what solutions to use, purchase, and to whom to market. A more controlled environment reduces alternative explanations for research findings and may have produced more internally valid results, but at the cost of less real-world application. A strength of this trial is that it was remotely conducted and therefore far-reaching. People of different race/ethnicities, social classes, and education levels participated from 43 out of the 50 states.

Participants came from across the United States but may not be representative of people with T1D in the country. Additionally, results may not generalize to specific racial/ethnic minority groups unaccounted for or underrepresented in this study. People with T1D in the United States are disproportionately non-Hispanic White (50%), followed by non-Hispanic Black (30%) and finally Hispanic (18%) and other race/ethnicities (5%) [39]. Our study sample was predominately non-Hispanic White (88%), but with much fewer non-Hispanic Black/Hispanic/Other (12%) racial/ethnic minority participants.

In addition to the study enrolling participants from all over the continental United States, recruitment, data collection, and analyses were also completed in only 6.4 months, saving time, money, and providing just-in-time results to decision makers. It generally takes 17 years to turn 14% of research findings into benefits for patients [40]. This trial strongly challenges how long it takes to conduct a randomized controlled trial and translate results into the real world.

Although the pragmatic and remote study design allowed for recruitment, data collection, and participation to occur in the context of everyday life, making it more convenient than trials requiring study visits at clinical trial sites, remoteness also meant relying on self-reported screening data, and medication and medical history. As a strength, engagement with One Drop (ie, protocol adherence) was objectively determined in real time. In addition, the primary outcome, hemoglobin A_1c_, was conveniently measured at home, but was processed at a central lab (ie, lab assays are the gold standard).

### Implications and Future Research

Better integration is the future of diabetes care. The closed-loop community highlights the desire for integrated tools (ie, people are hacking their insulin pumps and continuous glucose monitors to create their own artificial pancreas [41]). Results from this trial suggest that One Drop and an activity tracker may work better together than alone in helping people with T1D manage their health. To better understand the additive value of One Drop with and without an activity tracker among adults with T1D, future research should include four study arms (complete control, activity tracker only, One Drop only, One Drop and activity tracker) and randomize to condition. The current study was underpowered (N=95) to be a four-arm trial.

Data integration and control are key drivers of the initial and sustained use of consumer health technologies [36,42]. One Drop’s app is both integrated with activity trackers and allows users to have control over how they track their data and share it with their One Drop coach or a health care provider. Control, customization, and integration may influence longer-term use and benefit, and should be explored in future research.

## Appendix

Multimedia Appendix 1 CONSORT-eHEALTH (V 1.6.1).

## Abbreviations

ANCOVA: analysis of covariance
CDE: Certified Diabetes Educator
CONSORT: Consolidated Standards of Reporting Trials
FDA: Food and Drug Administration
HIPAA: Health Insurance Portability and Accountability Act
IRB: Institutional Review Board
ITT: intention to treat
PP: per protocol
T1D: type 1 diabetes
